# Non-invasive blood glucose estimation method based on the phase delay between oxy- and deoxyhemoglobin using visible and near-infrared spectroscopy

**DOI:** 10.1117/1.JBO.29.3.037001

**Published:** 2024-03-05

**Authors:** Tomoya Nakazawa, Rui Sekine, Masato Kitabayashi, Yu Hashimoto, Anna Ienaka, Keiji Morishita, Takeo Fujii, Masaki Ito, Fumie Matsushita

**Affiliations:** a Hamamatsu Photonics K.K., Hamamatsu, Japan; b Hamamatsu University, School of Medicine, Hamamatsu, Japan

**Keywords:** non-invasive blood glucose measurement, near-infrared spectroscopy, hemoglobin

## Abstract

**Significance:**

Many researchers have attempted to estimate blood glucose levels (BGLs) noninvasively using near-infrared (NIR) spectroscopy. However, the optical absorption change induced by blood glucose is weak in the NIR region and often masked by interference from other components such as water and hemoglobin.

**Aim:**

Instead of using direct optical absorption by glucose, this study proposes an index calculated from oxy- and deoxyhemoglobin signals that shows a good correlation with BGLs while using conventional visible and NIR spectroscopy.

**Approach:**

The metabolic index, which is based on tissue oxygen consumption, was derived through analytical methods and further verified and reproduced in a series of glucose challenge experiments. Blood glucose estimation units were prototyped by utilizing commercially available smart devices.

**Results:**

Our experimental results showed that the phase delay between the oxy- and deoxyhemoglobin signals in near-infrared spectroscopy correlates with BGL measured by a conventional continuous glucose monitor. The proposed method was also confirmed to work well with visible spectroscopy systems based on smartphone cameras. The proposed method also demonstrated excellent repeatability in results from a total of 19 oral challenge tests.

**Conclusions:**

This study demonstrated the feasibility of non-invasive glucose monitoring using existing photoplethysmography sensors for pulse oximeters and smartwatches. Evaluating the proposed method in diabetic or unhealthy individuals may serve to further increase its practicality.

## 1 Introduction

There is currently no curative treatment for type 1 and type 2 diabetes,^1^^,^^2^ patients need to monitor their blood glucose levels (BGLs) to prevent further progression of the disease. Although minimally invasive continuous glucose monitor (CGM) is becoming more widely used among diabetic patients,^3^ conventional self-monitoring blood glucose (SMBG) is still widely used because of its reliability, lower cost, and accuracy.^4^ However, SMBG requires a painful finger prick which sometimes results in low patient adherence.^5^ Therefore, reducing patient pain during blood glucose monitoring is an important issue. This is one of the reasons why non-invasive glucose monitoring is a research topic that has been studied for many years.^6^

Liakat et al.^7^ and Kottmann et al.^8^ used mid-infrared (MIR) light for glucose monitoring, focusing on the strong and distinct optical absorption of glucose at 9.5  μm. Although this is probably the most straightforward method, light sources, detectors, and other optical components used for MIR spectroscopy can be expensive and are still not easy to integrate into a portable device. Nielsen et al. and Pors et al.^9^^,^^10^ used Raman spectroscopy which has the advantage of low sensitivity to water and temperature changes, high specificity, and low cost. However, this method usually takes a certain amount of time for each measurement, and laser power and wavelength stability can sometimes be issues.^11^^,^^12^ Not to mention the fact that many researchers have used NIR spectroscopy which has the advantage of being inexpensive and easier to apply.^13^^,^^14^ However, the absorption peaks in the NIR region are less distinct and broader than those in the MIR region, making it difficult to filter out interference from other chemical components such as lipids and proteins.^11^ Cho et al.^15^ and Tang et al.^16^^,^^17^ developed the metabolic heat conformation (MHC) method, which utilizes the heat produced by metabolic oxidation which has a strong correlation with BGLs. Although this method also has the advantage of its feasibility and low cost, this technique suffers from interference due to environmental parameters.^18^ In addition, combinations of existing methods shown above with machine learning (ML) are also popular these days.^19^^,^^20^ However, ML methods sometimes fail to predict glucose levels because collecting large datasets with extensive and diverse blood glucose measurements can be challenging.

For the above reasons, there is still no practical non-invasive blood glucose monitoring device available for daily use. To address this problem, the authors applied data mining of photoplethysmography (PPG) data using visible and near-infrared light and found that there is a high correlation between the phase delay in deoxyhemoglobin compared to oxyhemoglobin and BGLs. The authors therefore propose in this paper a new index for non-invasive blood glucose measurement that utilizes practical and low-cost visible-NIR spectroscopy instead of expensive MIR spectroscopy, and is also fundamentally less sensitive to interference from environmental factors. The authors first explain the theory behind the metabolic index, which represents the degree of oxygen consumption in each cardiac cycle and is thought to be closely related to BGL from a cellular metabolism viewpoint. Their theoretical analysis is further validated by a short clinical study. Finally, the repeatability of the proposed index is confirmed.

## 2 Theory and Formulation

In this section, a new glucose level index is derived from basic near-infrared spectroscopy (NIRS) formulas.

### 2.1 Basic Formulas of NIRS

**Fig. 1:**
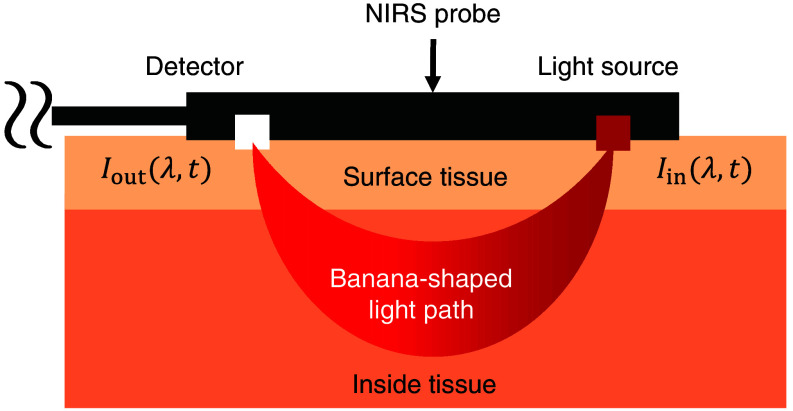
Schematic of NIRS measurement.

Figure 1 shows a schematic diagram of an NIRS measurement on a human body. Here, $I_{\text{in}}(\lambda ,t)$ and $I_{\text{out}}(\lambda ,t)$ are the incident and detected light intensities for the wavelength λ at time t, respectively. According to the modified Beer–Lambert law (MBLL), by using two different probe wavelengths and solving for the matrix calculation, the oxy- and deoxyhemoglobin NIRS signals $N_{{\mathrm{HbO}}_{2}}(t)$ and $N_{\mathrm{Hb}}(t)$ can be expressed as follows:^21^^–^^25^

$$N_{{\mathrm{HbO}}_{2}}(t)=\Delta [c_{{\mathrm{HbO}}_{2}}(t)\cdot L(t)]=c_{{\mathrm{HbO}}_{2}}(t)\cdot L(t)-c_{{\mathrm{HbO}}_{2}}(t_{0})\cdot L(t_{0}),$$ (1)

$$N_{\mathrm{Hb}}(t)=\Delta [c_{\mathrm{Hb}}(t)\cdot L(t)]=c_{\mathrm{Hb}}(t)\cdot L(t)-c_{\mathrm{Hb}}(t_{0})\cdot L(t_{0}),$$ (2)

where $c_{{\mathrm{HbO}}_{2}}(t)$ and $c_{\mathrm{Hb}}(t)$ are the molar concentrations of oxyhemoglobin and deoxyhemoglobin in the blood at time t, L(t) is the optical path length with respect to the time t, and the subscript 0 represents the initial condition, respectively. The process for formulating Eqs. (1) and (2) is described in detail in Sec. S1 of the Supplementary Material.

### 2.2 Derivation of Metabolic Index

**Fig. 2:**
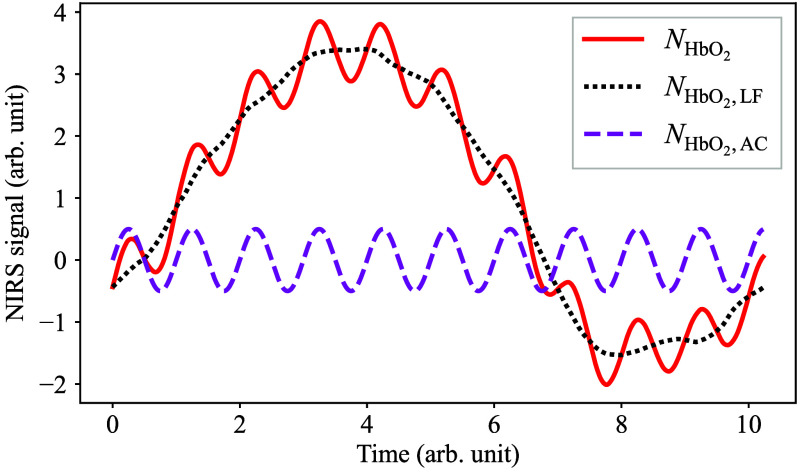
Conceptual diagram of NIRS signal decomposition into AC and LF components.

First, consider decomposing each component of Eqs. (1) and (2) into AC and low-frequency (LF) components. Figure 2 shows a conceptual diagram of NIRS signal decomposition into AC and LF signals. Here, it is assumed that heart rate variation is negligible, and the AC component is completely periodic as long as the time window is short enough. In this way, $N_{{\mathrm{HbO}}_{2}}(t)$ and $N_{\mathrm{Hb}}(t)$ can be rewritten as follows:

$$N_{{\mathrm{HbO}}_{2}}(t)=N_{{\mathrm{HbO}}_{2},\mathrm{LF}}(t)+N_{{\mathrm{HbO}}_{2},\mathrm{AC}}(t),$$ (3)

$$N_{\mathrm{Hb}}(t)=N_{\mathrm{Hb},\mathrm{LF}}(t)+N_{\mathrm{Hb},\mathrm{AC}}(t).$$ (4)

Here, the subscripts AC and LF indicate the AC and LF components of the corresponding physical quantity, respectively.

Similarly, $c_{{\mathrm{HbO}}_{2}}(t)$, $c_{\mathrm{Hb}}(t)$, and L(t) can be rewritten as combinations of AC and LF components as follows:

$$c_{{\mathrm{HbO}}_{2}}(t)=c_{{\mathrm{HbO}}_{2},\mathrm{LF}}(t)+c_{{\mathrm{HbO}}_{2},\mathrm{AC}}(t),$$ (5)

$$c_{\mathrm{Hb}}(t)=c_{\mathrm{Hb},\mathrm{LF}}(t)+c_{\mathrm{Hb},\mathrm{AC}}(t),$$ (6)

$$L(t)=L_{\mathrm{LF}}(t)+L_{\mathrm{AC}}(t).$$ (7)

By substituting Eqs. (5)–(7), Eqs. (1) and (2) are expressed as follows:

$$N_{{\mathrm{HbO}}_{2}}(t)=[c_{{\mathrm{HbO}}_{2},\mathrm{LF}}(t)+c_{{\mathrm{HbO}}_{2},\mathrm{AC}}(t)]\cdot [L_{\mathrm{LF}}(t)+L_{\mathrm{AC}}(t)]-c_{{\mathrm{HbO}}_{2}}(t_{0})\cdot L(t_{0}),$$ (8)

$$N_{\mathrm{Hb}}(t)=[c_{\mathrm{Hb},\mathrm{LF}}(t)+c_{\mathrm{Hb},\mathrm{AC}}(t)]\cdot [L_{\mathrm{LF}}(t)+L_{\mathrm{AC}}(t)]-c_{\mathrm{Hb}}(t_{0})\cdot L(t_{0}).$$ (9)

Furthermore, by expanding Eqs. (8) and (9) and by extracting the AC components

$$N_{{\mathrm{HbO}}_{2},\mathrm{AC}}(t)=c_{{\mathrm{HbO}}_{2},\mathrm{LF}}(t)\cdot L_{\mathrm{AC}}(t)+c_{{\mathrm{HbO}}_{2},\mathrm{AC}}(t)\cdot [L_{\mathrm{LF}}(t)+L_{\mathrm{AC}}(t)],$$ (10)

$$N_{\mathrm{Hb},\mathrm{AC}}(t)=c_{\mathrm{Hb},\mathrm{LF}}(t)\cdot L_{\mathrm{AC}}(t)+c_{\mathrm{Hb},\mathrm{AC}}(t)\cdot [L_{\mathrm{LF}}(t)+L_{\mathrm{AC}}(t)],$$ (11)

can be derived. Here it is assumed that $N_{{\mathrm{HbO}}_{2},\mathrm{AC}}(t)$ and $N_{\mathrm{Hb},\mathrm{AC}}(t)$ are completely periodic, and the average over a cycle becomes zero.

Next, to simplify Eqs. (10) and (11), the following additional constraints are introduced:

$$c_{{\mathrm{HbO}}_{2},\mathrm{LF}}(t)+c_{\mathrm{Hb},\mathrm{LF}}(t)=c_{0}=\mathrm{const.},$$ (12)

$$c_{{\mathrm{HbO}}_{2},\mathrm{AC}}(t)+c_{\mathrm{Hb},\mathrm{AC}}(t)=0,$$ (13)

$$L_{\mathrm{LF}}(t)\gg |L_{\mathrm{AC}}(t)|,\;L(t)=L_{\mathrm{LF}}(t)+L_{\mathrm{AC}}(t)\approx L_{\mathrm{LF}}(t),$$ (14)

where $c_{0}$ is a constant and corresponds to the total hemoglobin concentration.

Here, Eq. (12) means that the molar concentration of the total hemoglobin in the blood is conserved within a limited measurement period, and Eq. (13) means that the increase in $c_{{\mathrm{HbO}}_{2},\mathrm{AC}}(t)$ is equal to the decrease in $c_{\mathrm{Hb},\mathrm{AC}}(t)$ and vice versa, and Eq. (14) means that the oscillation amplitude of the optical path length is much smaller than the LF component of the optical path length.

**Fig. 3:**
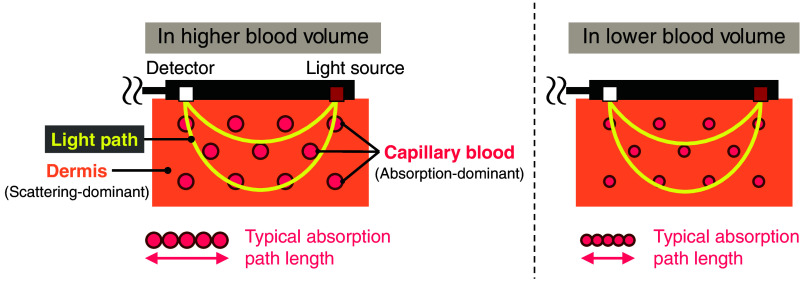
Schematic of the change in the optical path length in correlation with the blood volume.

In many prior studies, the optical path length L(t) has been considered constant, leading to the interpretation of oxy- and deoxyhemoglobin NIRS signals as volume concentrations in the light-probed region. However, this study assumes that the optical path length L(t) pulsates and changes slowly in correlation with the blood flow and heartbeat and that the oxy- and deoxyhemoglobin concentration $c_{{\mathrm{HbO}}_{2}}(t)$ and $c_{\mathrm{Hb}}(t)$ are molar concentrations in the blood. To explain the validity of these assumptions, Fig. 3 illustrates the schematic of the change in the optical path length.

Since hemoglobin is the most dominant light absorber in the NIR region, light absorption by the dermis can be ignored and the dermis can be treated as a scattering-dominant component. Therefore, the typical absorption path length can be expressed as the total length of capillary blood probed by the light. Based on this idea, the optical path length varies along with changes in both blood flow and heartbeat due to the variability in the capillary diameter. However, the assumption that the scattering change due to blood flow change and heartbeat is negligible forms the basis of this premise. The dermis meets this premise by taking into account the capillary density.^26^

By using Eqs. (12) and (13) in Eqs. (10) and (11), the following relationship can be derived:

$$L_{\mathrm{AC}}(t)=\frac{1}{c_{0}}[N_{{\mathrm{HbO}}_{2},\mathrm{AC}}(t)+N_{\mathrm{Hb},\mathrm{AC}}(t)].$$ (15)

Then, by substituting Eq. (15) into the first term on the right side of Eq. (11) and by simplifying with respect to $c_{\mathrm{Hb},\mathrm{AC}}(t)$, the following can be derived:

$$c_{\mathrm{Hb},\mathrm{AC}}(t)=\frac{1}{L(t)}\cdot \frac{c_{{\mathrm{HbO}}_{2},\mathrm{LF}}(t)N_{\mathrm{Hb},\mathrm{AC}}(t)-c_{\mathrm{Hb},\mathrm{LF}}(t)N_{{\mathrm{HbO}}_{2},\mathrm{AC}}(t)}{c_{{\mathrm{HbO}}_{2},\mathrm{LF}}(t)+c_{\mathrm{Hb},\mathrm{LF}}(t)},$$ (16)

$$=\frac{1}{L(t)}\cdot \{{\mathrm{StO}}_{2}(t)\cdot N_{\mathrm{Hb},\mathrm{AC}}(t)-[1-{\mathrm{StO}}_{2}(t)]\cdot N_{{\mathrm{HbO}}_{2},\mathrm{AC}}(t)\},$$ (17)

where ${\mathrm{StO}}_{2}(t)$ is the tissue hemoglobin oxygen saturation^27^ defined as follows:

$${\mathrm{StO}}_{2}(t)=\frac{c_{{\mathrm{HbO}}_{2},\mathrm{LF}}(t)}{c_{{\mathrm{HbO}}_{2},\mathrm{LF}}(t)+c_{\mathrm{Hb},\mathrm{LF}}(t)}.$$ (18)

Since $c_{\mathrm{Hb},\mathrm{AC}}(t)$ corresponds to the oxygen consumption during each heartbeat cycle generated by cell respiration, it is hypothesized that the metabolic levels in the human body can be estimated from the amplitude of $c_{\mathrm{Hb},\mathrm{AC}}(t)$.^28^

Here, within a limited time window, the AC-NIRS signal $N_{{\mathrm{HbO}}_{2},\mathrm{AC}}(t)$ and $N_{\mathrm{Hb},\mathrm{AC}}(t)$ can be approximated by sinusoids with slowly varying amplitudes by neglecting gradual changes in heart rate as follows:

$$N_{{\mathrm{HbO}}_{2},\mathrm{AC}}(t)=A_{{\mathrm{HbO}}_{2}}(t)\sin\;\omega t,$$ (19)

$$N_{\mathrm{Hb},\mathrm{AC}}(t)=A_{\mathrm{Hb}}(t)\sin\;\omega t,$$ (20)

where ω is the angular frequency of the heartbeat, and $A_{{\mathrm{HbO}}_{2}}(t)$ and $A_{\mathrm{Hb}}(t)$ are the slowly varying amplitudes of the respective AC-NIRS signals whose time scales are much larger than the oscillation cycle 2π/ω and can be treated as nearly constant within a limited measurement period, such as the $N_{{\mathrm{HbO}}_{2},\mathrm{AC}}$ curve in Fig. 2. Considering that the arterial blood is the dominant factor for the pulsating AC components,^29^ the instantaneous arterial oxygen saturation ${\mathrm{SaO}}_{2}(t)$ can be expressed as follows:

$${\mathrm{SaO}}_{2}(t)=\frac{A_{{\mathrm{HbO}}_{2}}(t)}{A_{{\mathrm{HbO}}_{2}}(t)+A_{\mathrm{Hb}}(t)}.$$ (21)

Here, by properly selecting a measurement location on the body where the arteries connect to the cellular respiration site, and by selecting the distance between the detector and the light source of the NIRS probe to allow for a shallow penetration depth of the light path, the following equation can be applied:

$${\mathrm{StO}}_{2}(t)\approx {\mathrm{SaO}}_{2}(t).$$ (22)

In this study, e.g., the capillary-rich fingertip is identified as one of the suitable sites, and a relatively short detector-light source distance should also be utilized to prevent reaching bone depth. Then, by substituting Eqs. (19)–(22), Eq. (17) becomes

$$c_{\mathrm{Hb},\mathrm{AC}}(t)=\frac{1}{L(t)}\cdot \frac{A_{{\mathrm{HbO}}_{2}}(t)\cdot N_{\mathrm{Hb},\mathrm{AC}}(t)-A_{\mathrm{Hb}}(t)\cdot N_{{\mathrm{HbO}}_{2},\mathrm{AC}}(t)}{A_{{\mathrm{HbO}}_{2}}(t)+A_{\mathrm{Hb}}(t)}=0.$$ (23)

Here, Eq. (23) means that pulsation in deoxyhemoglobin (or oxyhemoglobin) concentration does not exist and is counterintuitive. To resolve this contradiction, consider modifying Eq. (20) as

$$N_{\mathrm{Hb},\mathrm{AC}}(t)=A_{\mathrm{Hb}}(t)\sin[\omega t-\Delta \theta (t)],$$ (24)

where Δθ(t) is a slowly varying and sufficiently small phase delay.Equation (24) means that $N_{\mathrm{Hb},\mathrm{AC}}(t)$ has a small phase delay with respect to $N_{{\mathrm{HbO}}_{2},\mathrm{AC}}(t)$. The validity of this assumption will be verified by experiments in later sections.

Then, by substituting Eqs. (19) and (24), Eq. (23) becomes as follows:

$$c_{\mathrm{Hb},\mathrm{AC}}(t)=\frac{1}{L(t)}\cdot \frac{A_{{\mathrm{HbO}}_{2}}(t)\cdot A_{\mathrm{Hb}}(t)}{A_{{\mathrm{HbO}}_{2}}(t)+A_{\mathrm{Hb}}(t)}\cdot \{\sin[\omega t-\Delta \theta (t)]-\sin(\omega t)\},$$ (25)

$$=-\frac{2\;\sin[\Delta \theta (t)/2]}{L(t)}\cdot \frac{A_{{\mathrm{HbO}}_{2}}(t)\cdot A_{\mathrm{Hb}}(t)}{A_{{\mathrm{HbO}}_{2}}(t)+A_{\mathrm{Hb}}(t)}\cdot \cos[\omega t-\frac{\Delta \theta (t)}{2}],$$ (26)

$$\approx -\frac{\Delta \theta (t)}{L(t)}\cdot \frac{A_{{\mathrm{HbO}}_{2}}(t)\cdot A_{\mathrm{Hb}}(t)}{A_{{\mathrm{HbO}}_{2}}(t)+A_{\mathrm{Hb}}(t)}\cdot \cos[\omega t-\frac{\Delta \theta (t)}{2}],$$ (27)

$$=-\frac{A_{{\mathrm{HbO}}_{2}}(t)+A_{\mathrm{Hb}}(t)}{L(t)}\cdot {\mathrm{SaO}}_{2}(t)\cdot [1-{\mathrm{SaO}}_{2}(t)]\cdot \Delta \theta (t)\cdot \cos[\omega t-\frac{\Delta \theta (t)}{2}].$$ (28)

Then, assuming that $A_{{\mathrm{HbO}}_{2}}(t)$, $A_{\mathrm{Hb}}(t)$, L(t), and Δθ(t) change on a much slower time scale than 2π/ω, the oscillation amplitude of Eq. (28). $C_{\mathrm{Hb},\mathrm{AC}}(t)$ is obtained as follows:

$$C_{\mathrm{Hb},\mathrm{AC}}(t)=\frac{A_{{\mathrm{HbO}}_{2}}(t)+A_{\mathrm{Hb}}(t)}{L(t)}\cdot {\mathrm{SaO}}_{2}(t)\cdot [1-{\mathrm{SaO}}_{2}(t)]\cdot |\Delta \theta (t)|.$$ (29)

In addition, from Eqs. (15), (19), and (24), the following relation is derived:

$$A_{{\mathrm{HbO}}_{2}}(t)+A_{\mathrm{Hb}}(t)\approx c_{0}L_{\mathrm{AC},\text{amplitude}}(t),$$ (30)

where $L_{\mathrm{AC},\text{amplitude}}(t)$ is the slowly varying oscillation amplitude of $L_{\mathrm{AC}}(t)$.

Finally, by substituting Eq. (30) into Eq. (29)

$$C_{\mathrm{Hb},\mathrm{AC}}(t)\approx c_{0}\cdot \frac{L_{\mathrm{AC},\text{amplitude}}(t)}{L(t)}\cdot \mathrm{MI}(t),$$ (31)

where MI(t) is a dimensionless metabolic index defined as follows:

$$\mathrm{MI}(t)={\mathrm{SaO}}_{2}(t)\cdot [1-{\mathrm{SaO}}_{2}(t)]\cdot |\Delta \theta (t)|.$$ (32)

Looking at Eq. (31), the pulsation amplitude of deoxyhemoglobin $C_{\mathrm{Hb},\mathrm{AC}}(t)$ increases as the total hemoglobin concentration $c_{0}$, the relative AC optical path length amplitude $L_{\mathrm{AC},\text{amplitude}}(t)/L(t)$, and the absolute phase delay |Δθ(t)| increase. Here, $L_{\mathrm{AC},\text{amplitude}}(t)/L(t)$ is supposed to be a value similar to the perfusion index (PI), which is widely used in the field of pulse oximetry to evaluate signal quality.^30^ On the other hand, $C_{\mathrm{Hb},\mathrm{AC}}(t)$ decreases with increasing arterial oxygen saturation ${\mathrm{SaO}}_{2}(t)$. In particular, $C_{\mathrm{Hb},\mathrm{AC}}(t)$ decreases to zero as ${\mathrm{SaO}}_{2}(t)$ reaches 100%. This change indicates that aerobic respiration does not occur in areas with 100% oxygen saturation, such as in arteries. This change also highlights the importance of carefully selecting the measurement location on the body.

Since the $c_{0}$ for each specific subject does not change significantly unless there is drastic dehydration or anemia, and since $L_{\mathrm{AC},\text{amplitude}}(t)/L(t)$ should also be nearly constant without exercise and temperature change of the measurement portion, MI(t) is the dominant factor in oxygen consumption which should strongly correlate with the body metabolism in a resting state.

Furthermore, Eq. (32) reveals that every term on the right-hand side is derived solely from the AC component of the oxy- and deoxyhemoglobin NIRS signals $N_{{\mathrm{HbO}}_{2},\mathrm{AC}}(t)$ and $N_{\mathrm{Hb},\mathrm{AC}}(t)$, without incorporating the LF component. This fact implies that the drift in the light source intensity does not significantly impact MI(t) since the light source drift has a negligible effect on the AC component of the calculated NIRS signal in the MBLL.

In the following sections, an attempt to investigate the correlation between the metabolic index MI and blood glucose is explained through a series of experiments.

## 3 Materials and Methods

In this section, the proposed MI-based BGL estimation method is investigated using two types of prototype devices.

### 3.1 Evaluation Using a Smartwatch-Based Prototype

A conceptual validation of the proposed MI method through a smartwatch-based prototype is presented in this section.

**Fig. 4:**
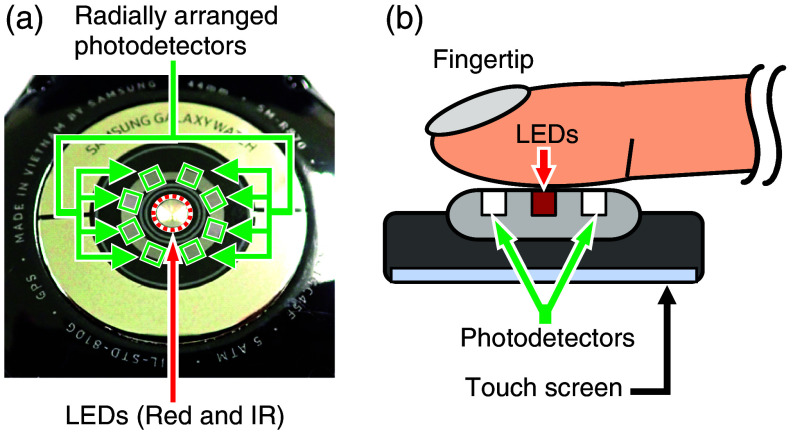
(a) Rear view of the smartwatch experimental unit. (b) Schematic diagram of the measurement using a smartwatch worn on a fingertip.

In the initial evaluation, the Samsung Galaxy Watch 4 44 mm (SM-R870) was used as the experimental unit. Figure 4(a) shows the rear view of the SM-R870. Red and IR LEDs are located in the center, and their center wavelengths are ∼650 and 930 nm, respectively. Eight photodetectors are arranged radially around the LEDs. The sampling frequency of the red and IR LED signals is 100 Hz. Here, the experimental device has no hardware modification from its commercially available state. Figure 4(b) shows a schematic of the measurement. Originally, the LEDs and photodetectors in the smartwatch were designed to be used while wearing it on the wrist. However, due to its capillary density, the human wrist is not the best position for capturing a strong pulse wave signal. Therefore, instead of wearing it on the wrist, the smartwatch was wrapped on a finger pad.

Functions to retrieve raw photodetector data from Galaxy Watch devices require the Samsung Privileged Health SDK, which is not publicly available. Therefore, the Samsung Partner App Program was used to gain access to the smartwatch functions needed for this experiment.

#### 3.1.1 Experimental protocol

**Fig. 5:**
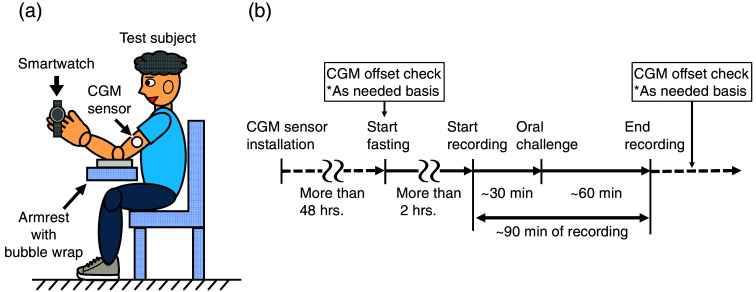
(a) Schematic diagram of the clinical trial setup. (b) Clinical trial procedure.

Figure 5(a) shows the schematic of the clinical test setup. A protocol was established while referencing factors that can affect reading values in the case of a pulse oximeter.^31^ Healthy and non-diabetic subjects were asked to sit still during the experiment with the smartwatch resting on their finger pad to reduce fluctuations in peripheral blood flow caused by minor changes in posture and local blood pressure. Subjects were also asked to rest their elbow on the smartwatch-wearing side on an armrest. Bubble wrap was placed on the armrest to relieve pressure and prevent hypoperfusion. In addition, before starting the measurement, subjects held a hand warmer for a few minutes to ensure adequate peripheral blood flow.

Figure 5(b) shows the typical course of the clinical test. A CGM device (Abbot, FreeStyle Libre^®^) was used as a reference for the BGLs. Since it is reported that CGM sensors do not exhibit their best performance during the first few days after installation,^32^ CGM sensors were installed at least 2 days before the experiments for aging purposes. In addition, to correct for the systematic offset and delay specific to each CGM sensor, SMBG strips (Abbot, FreeStyle Precision^®^) were used as needed before and after data recording by the smartwatch. To prevent possible distortion of the PPG signal due to body movements resulting from SMBG usage, no SMBG strips were used during the recording period. Data recording then started after at least 2 h of fasting, oral challenges were given about 30 min after the start of recording, and data recording continued about 60 min after the oral challenges. As a result, ∼90  min of PPG data were collected in each data recording.

Sugar-containing carbonated beverages (The Coca-Cola Company, Coca-Cola^®^ Original 350 mL) and glucose-containing jelly beverages (Morinaga, in Jelly^®^ Energy) were used for oral challenges. Typically, a special, highly concentrated glucose solution is used for this type of oral challenge test. However, since this is a concept test, more human-friendly oral challenges have been adapted this time. In addition, sugar-free carbonated beverages (The Coca-Cola Company, Coca-Cola^®^ Zero Sugar 350 mL) and non-carbonated bottled water were used for sugar-free oral challenges in the control experiments. Care was taken not to make all oral challenges excessively cold since this would reduce peripheral blood flow due to lowering of the body temperature. Finally, the main nutritional values of the oral challenges are listed in Table 1.

**Table 1:** Key nutritional facts of oral challenges per serving.

Item	Coca-Cola^®^ Original	In Jelly^®^ Energy	Coca-Cola^®^ Zero Sugar	Bottled water
Serving size	350 mL	180 g	350 mL	500 mL
Calories (kCal)	140	180	0	0
Fat (g)	0	0	0	0
Carbohydrate (g)	39	45	0	0
Protein (g)	0	0	0	0

All clinical trials described in this paper were conducted in accordance with the Clinical Trials Act of the Ministry of Health, Labor and Welfare of Japan, published on the basis of the Declaration of Helsinki, and were approved by the Ethical Committee of Hamamatsu Photonics K. K. Informed consent was obtained from all subjects before measurements were performed. All clinical tests were performed under the supervision of the co-author with a medical license.

#### 3.1.2 Data processing

**Fig. 6:**
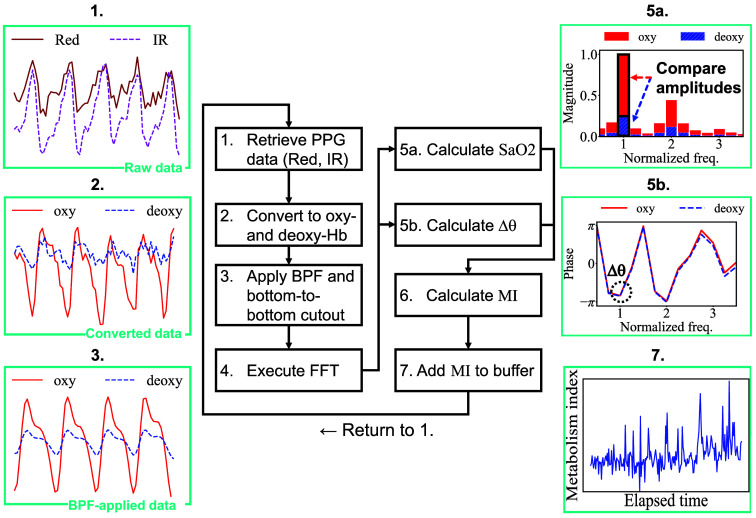
Process flowchart of the metabolic index MI measurement, and visual explanations of several calculation steps.

Figure 6 shows the process flowchart for the metabolic index MI measurement used in this study and visual explanations of several operational steps. First, raw PPG data from red and IR LEDs were retrieved and accumulated to a certain data length.

Next, the raw PPG data were converted to the NIRS signals $N_{{\mathrm{HbO}}_{2}}(t)$ and $N_{\mathrm{Hb}}(t)$ according to the steps shown in Sec. 2.1. Here, the transmitted light intensity is assumed to be proportional to the PPG signal, and the extinction coefficients of oxyhemoglobin and deoxyhemoglobin compiled by Scott Prahl^33^ were used for the calculation.

A second-order Butterworth band-pass filter (BPF) was then applied to each NIRS signal to remove unnecessary high-frequency components and LF components due to respiratory cycles. Here, the passband, the maximum loss in the passband, and the minimum attenuation in the stopband were 0.8 to 10 Hz, 3 dB, and 10 dB, respectively. After applying the BPF, the excess end portions in each waveform were trimmed so that the first and last points of the data corresponded to the beginning and end of the pulse wave.

A fast Fourier transform (FFT) was then applied to the trimmed NIRS signals. Each trimmed signal was resampled to make the data length the smallest power of two greater than or equal to the original length, and the Hamming window was selected for a window function.

Then, ${\mathrm{SaO}}_{2}(t)$ and Δθ(t) were calculated from the previously mentioned FFT spectra. First, by comparing the magnitude of each main peak whose frequency corresponds to the heart rate, ${\mathrm{SaO}}_{2}(t)$ can be calculated according to Eq. (21). Second, Δθ(t) can be calculated by comparing the FFT phase at the main peak.

Finally, the metabolic index MI(t) was calculated according to Eq. (32), and the calculated MI(t) was successively appended to a data buffer.

**Fig. 7:**
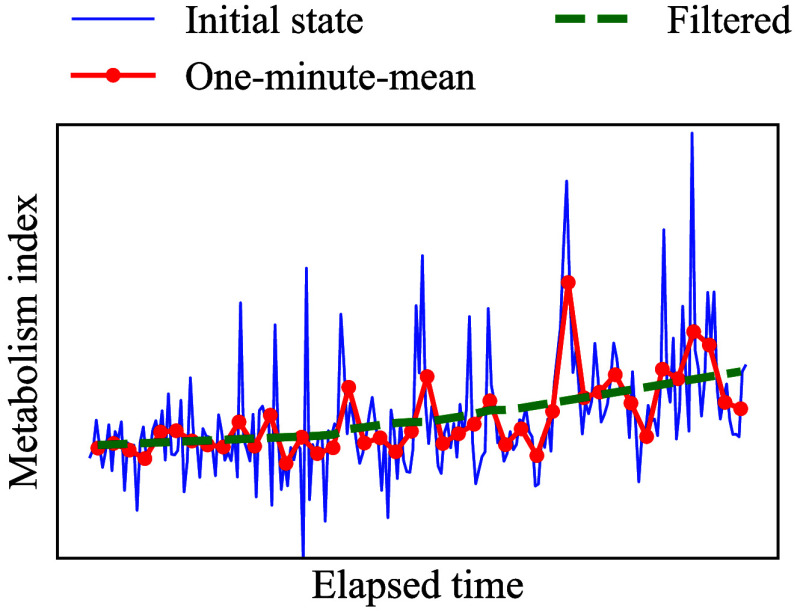
Visual explanation of the post-processing of the calculated MI values.

After the recording was completed, a series of recorded MI data were further processed as follows. Figure 7 shows a visual explanation of the post-processing of the calculated MI values. First, the MI values were divided into 1-min chunks. Then, the mean within each chunk was calculated after removing obvious outliers. Finally, the Savitzky-Golay filter was applied to the one-minute averages to smooth out fluctuations due to body motion, measurement errors, etc. Here, the polynomial order and window length for the Savitzky-Golay filter were 1 and 29, respectively. Although Fig. 7 contains outliers due to the above reasons, under ideal conditions, three to five data sets each consisting of about four heartbeat signals, are sufficient to derive a stable and instantaneous average MI value. In this ideal case, the total measurement time required is ∼7.2 to 20 s assuming that the normal heart rate of adults is 60 to 100 bpm.

#### 3.1.3 Results from the smartwatch-based prototype

**Fig. 8:**
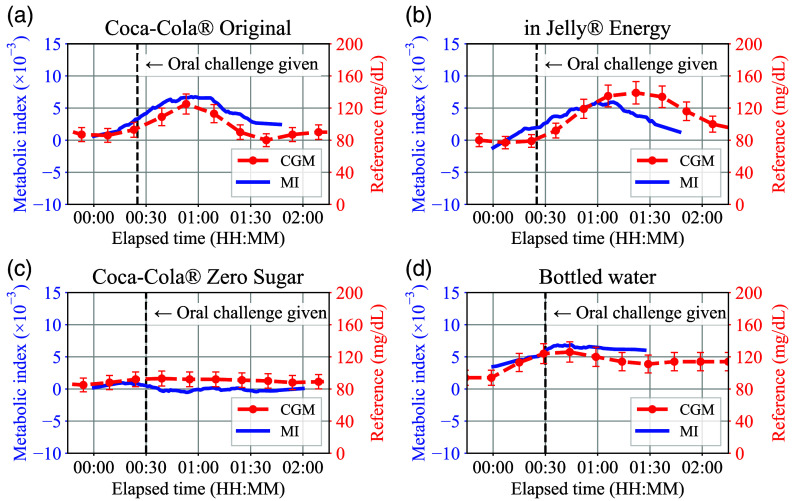
(a)–(d) Evaluation results of the smartwatch-based prototype for the different oral challenge types.

Figures 8(a)–8(d) show the evaluation results of the smartwatch-based prototype according to different types of oral challenges for the same male subject who has no underlying health conditions. Here, the vertical dashed line in each plot indicates the time when the oral challenge was given to the subject, and the 10% vertical error bar range was applied to the reference CGM values considering the typical accuracy of the sensor.^34^

**Fig. 9:**
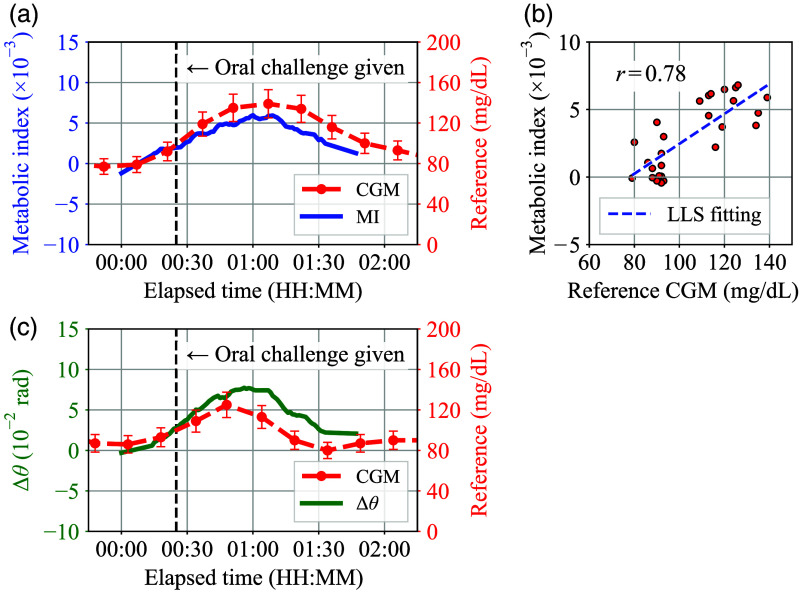
(a) Delay-compensated plot of Fig. 8(b). (b) Scatter plot of MI values versus reference CGM values generated from the data from Figs. 8(a), 8(c), and 8(d) and Fig. 9(a). (c) Transition of Δθ(t) in Fig. 8(a).

In Fig. 8(a), the metabolic index MI agreed well with the reference glucose concentration transition induced by the sugary drink ingestion. On the other hand, in Figs. 8(c) and 8(d), both MI and reference values were maintained nearly flat during each oral challenge test. Obviously, the sugar-free drink does not affect the subject’s BGL that much. However, the finding that MI values did not change as much during the sugar-free oral challenge tests suggests that MI is not simply a reflection of the body’s hydration status. At the same time, judging from the results that both carbonated and non-carbonated sugar-free drinks showed similar flat MI trends, the result in Fig. 8(a) is unlikely to be due to the carbonation status of the oral challenge. Regarding Fig. 8(b), an offset of about 15 min that can be seen between MI and CGM is likely due to the lag between blood glucose and interstitial fluid (ISF) glucose. In general, 15 min of CGM sensor delay is a bit large compared to the typical delay reported by the manufacturer. However, a previous study^35^ shows that CGM delay can reach 15 min. Then, by compensating for 15 min of offset in Fig. 8(b), the plot can be modified as in Fig. 9(a). In this case, the MI also follows the up and down trend of the CGM well, in the same way as seen in Fig. 8(a). The proposed MI method response time can be estimated as 5 to 15 min from Figs. 8(a)–8(d) considering that the CGM device time delay is 5 to 15 min and the MI exhibited a similar trend.

By compiling the plot data from Figs. 8(a), 8(c), 8(d), and 9(a), a scatter plot Fig. 9(b) can be obtained. Here, each numerical data of MI corresponding to each CGM data point has been calculated by interpolation. In Fig. 9(b), the correlation coefficient r calculated by the linear least squares (LLS) fitting reached 0.78, which indicates that the proposed metabolic index MI has a strong correlation with BGL.

In addition, the arterial oxygen saturation ${\mathrm{SaO}}_{2}(t)$ remained between 90±2% during a series of experiments, which means that ${\mathrm{SaO}}_{2}(t)\cdot [1-{\mathrm{SaO}}_{2}(t)]$ remained between 0.07 and 0.11 in Eq. (32), and the maximum to minimum ratio of ${\mathrm{SaO}}_{2}(t)\cdot [1-{\mathrm{SaO}}_{2}(t)]$ was at most ∼1.4. Here, the decrease in ${\mathrm{SaO}}_{2}(t)$ from its normal range is attributed to the measurement section where ${\mathrm{SaO}}_{2}(t)$ approaches ${\mathrm{StO}}_{2}(t)$.^36^

Therefore, the increase in the metabolic index MI was largely due to the change in Δθ(t): the phase delay between oxy- and deoxyhemoglobin. For reference, the Δθ(t) transition in the experiment of Fig. 8(a) is shown in Fig. 9(c). In this study, the results consistently showed that Δθ(t) was positive during each oral challenge test, indicating that the oxyhemoglobin NIRS signal $N_{{\mathrm{HbO}}_{2}}(t)$ consistently precedes the deoxyhemoglobin NIRS signal $N_{\mathrm{Hb}}(t)$. The MI trend is therefore almost identical to the non-absolute Δθ(t) trend. For this reason, only the MI trends are plotted in this paper to evaluate the proposed method, rather than separately plotting Δθ(t) and ${\mathrm{SaO}}_{2}(t)$.

Thus, the validity of the assumptions in Eq. (24) is verified.

### 3.2 Evaluation Using a Smartphone Camera-Based Prototype

The conceptual validation of the proposed MI method has been confirmed in Sec. 3.1. This section examines the reproducibility of the proposed method using a smartphone-based prototype.

Although the number of smartwatches is currently increasing worldwide, the penetration rate of smartwatches still has not reached that of smartphones. The utilization of smartphones as blood glucose monitoring devices has the potential to drastically expand the accessibility of the proposed MI method, given the global penetration of smartphones. Moreover, the active area and sensitivity of photodiodes in smartwatches are the minimum necessary due to spatial constraints, the main processors of smartwatches are less powerful, and the flexibility in designing smartwatch applications is limited compared to those of smartphones. In addition, several previous studies on smartphone camera-based non-invasive blood glucose monitoring have recently been reported.^37^^,^^38^

For the above reasons, the smartphone camera-based prototype is newly introduced for the repeatability test instead of the smartwatch-based prototype.

**Fig. 10:**
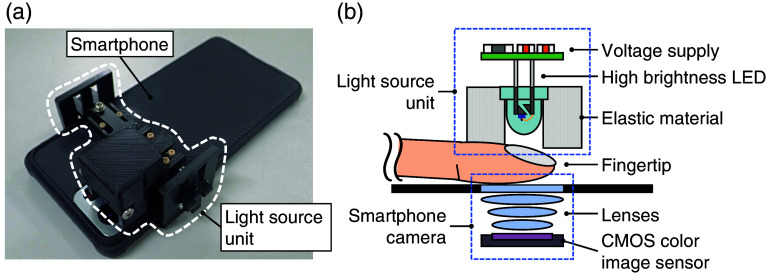
(a) Overview of the smartphone camera-based prototype. (b) Internal structure diagram of the light source unit.

Figure 10(a) shows an overview of a smartphone-based prototype developed for this research. The prototype consists of a smartphone and a light source unit mounted over the main camera module of the smartphone. Figure 10(b) shows the internal structure of the light source unit. Here, Samsung Galaxy S21 Ultra 5G (SC-52B) was selected as the smartphone and its wide-angle camera has a pixel count of 108 megapixels.

**Fig. 11:**
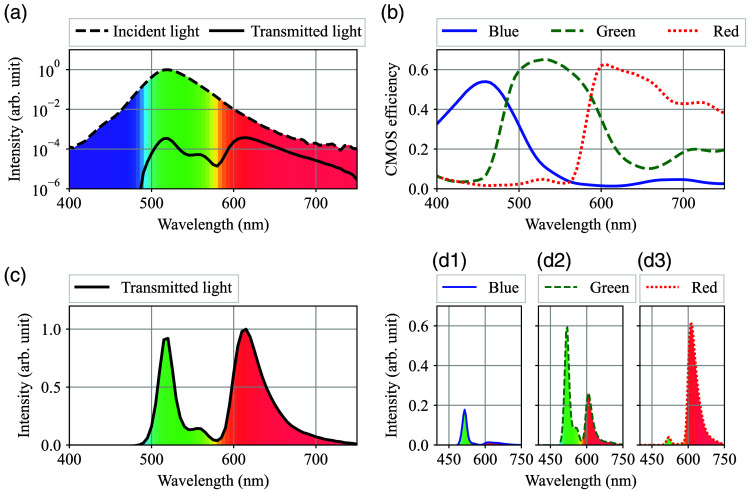
(a) Typical spectrum of a green LED and its transmitted spectrum after passing through a fingertip in log Y scale. (b) Typical efficiency curves of an RGB CMOS camera. (c) The normalized transmitted spectrum of the green LED after passing through a fingertip (d1)–(d3) Light intensity of the transmitted light perceived by each RGB sensor of the CMOS camera.

In addition, a high-brightness green LED OptoSupply OSG59L5B61Y was selected as the light source. The reason why green LED was chosen instead of white LED is described in Figs. 11(a)–11(c) and 11(d1)–11(d3). Figure 11(a) shows the typical spectrum of the green LED and its transmission spectrum after the subject’s index finger. Note that the y-axis is a logarithmic scale, and the spectra have been filled in with a color corresponding to each wavelength for visual support. Because LEDs have a broad spectrum compared to laser diodes (LDs), the green LED has a slight amount of reddish components in the tails of its spectrum.

Next, Fig. 11(c) shows the transmitted spectrum of the green LED on a linear scale. Due to the strong absorption by oxy- and deoxyhemoglobin around 550 and 400 nm, the initial Gaussian-like spectrum of the green LED is transformed into a two-hump shape. Then, Fig. 11(b) shows the typical quantum efficiency of an RGB color CMOS camera. Since the quantum efficiency data of the Samsung Galaxy S21 Ultra 5G smartphone camera was not available, data from a compact scientific CMOS color digital camera from Thorlabs^39^ was used as a typical value instead. Finally, by multiplying the quantum efficiency by the transmitted light, the light intensity spectrum perceived by the blue, green, and red CMOS sensors can be obtained as shown in Figs. 11(d1)–11(d3), respectively. Looking at Figs. 11(d1) and 11(d3), it can be seen that each spectrum is composed of almost a single prominent peak. Therefore, by combining the light intensity data from blue and red CMOS sensors, a kind of visible spectroscopy for the human fingertip can be configured.

**Fig. 12:**
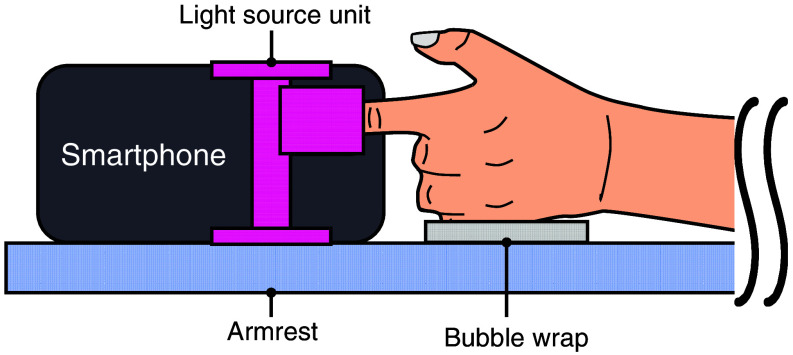
Test setup for the smartphone camera prototype.

The experimental protocol for the smartphone prototype largely followed that described in Sec. 3.1.1. Figure 12 illustrates the test setup for the smartphone prototype. The index finger of a healthy, non-diabetic subject with no underlying health conditions was placed between the light source unit and the smartphone camera, and a bubble wrap sheet was placed against the hypothenar to relieve pressure and prevent hypoperfusion. Since the smartphone prototype utilizes a transmissive optical configuration, the distance between the light source and detector was optimized by adjusting the fingertip insertion depth. The autofocus, auto exposure, and auto white balance functions of the smartphone camera were disabled. In addition, the camera aperture and frame rate were kept constant throughout the clinical trial. The camera sensitivity was kept to approximately the same value.

With regard to oral challenges, Coca-Cola^®^ Original or in Jelly^®^ Energy was administered to the subject and no sugar-free challenge was used, and oral challenges were administered between 15 and 30 min after the start of each test. Furthermore, stricter expiration date controls were also applied to the CGM sensors. In addition to the first two days for aging, the CGM sensors were not used for clinical testing during the last two days of their specified expiration date to improve performance.^32^

The data analysis process was the same as explained in Sec. 3.1.2 and Figs. 6 and 7 except for the method for acquiring raw PPG data. Instead of red and IR PPG data, the average pixel values of blue and red CMOS data were used. Note that camera images captured by the video stream function of Android OS are formatted in YUV420, so each YUV420 image must be converted to RGB format. Furthermore, since the extinction coefficient dataset of oxy- and deoxyhemoglobin used in Sec. 3.1.2 did not work properly in the case of pseudo-visible spectroscopy by the smartphone camera prototype, the extinction coefficient matrix was manually optimized to obtain a smartphone prototype result comparable to that of the smartwatch prototype. Finally, for the BPF and Savitzky–Golay filters, the same parameters were used as in Sec. 3.1.2.

#### 3.2.1 Results from the smartphone camera-based prototype

**Fig. 13:**
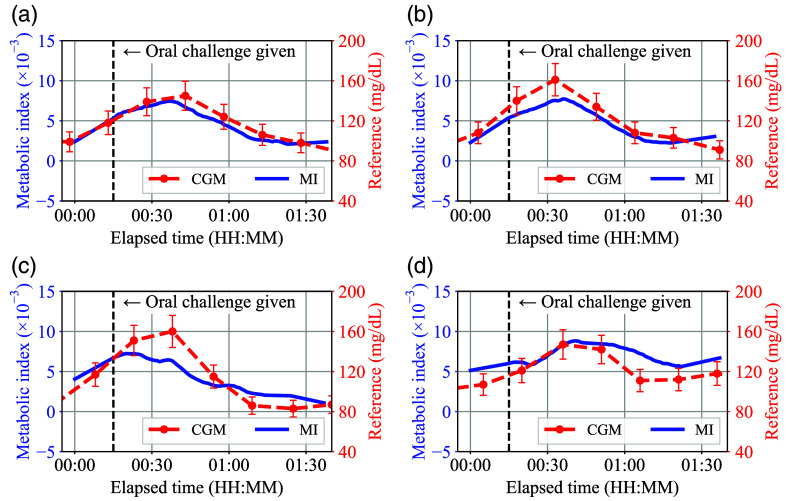
(a) and (b) Typical evaluation results of the smartphone-based prototype with a good correlation to the reference. (c) and (d) Typical results with a moderate correlation to the reference.

The repeatability test of the smartphone-based prototype was repeated a total of 19 times on a single male subject with no underlying health conditions. Figure 13 shows four typical evaluation results of the smartphone-based prototype from the 19 tests. Here, the test results are plotted in the same way as in Fig. 8, and the CGM delays were adjusted by using the sensor-specific constant value of each sensor, which was determined through comparison with SMBG readings during the preparation period. Figures 13(a) and 13(b) are examples of good correlation with the reference CGM, while Figs. 13(c) and 13(d) are examples of moderate correlation. In each of Figs. 13(a)–13(d), it can be seen that the metabolic index MI responded to the oral challenges and followed the CGM trends. Here, it should be noted that the trend of MI in each of Figs. 13(a)–13(c) appears to increase even under fasting conditions, which can be attributed to the smoothing effect of the Savitzky–Golay filter.

**Fig. 14:**
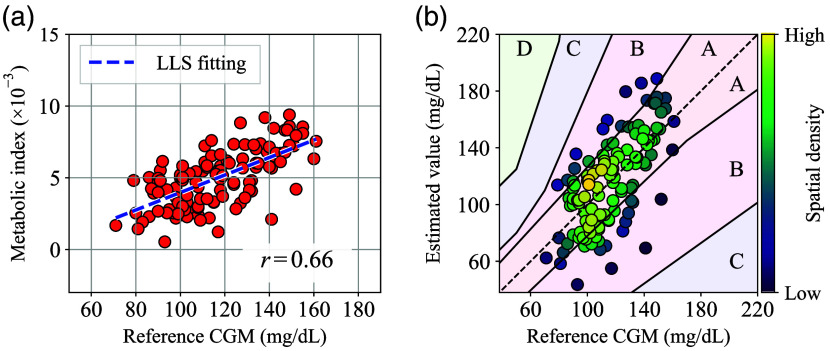
(a) Scatter plot of metabolic index MI values versus reference CGM values generated from repeatability test results. (b) Perkes error grid for type 1 diabetes generated from the repeatability test results.

Figure 14(a) shows a scatter plot of the metabolic index MI versus the reference CGM made from the repeatability test results. Here, each MI numerical data corresponding to each CGM data point was calculated by interpolation in the same way as in Fig. 9(b), and the correlation coefficient obtained by LLS fitting was 0.66. Although this correlation coefficient value r is not as high as that obtained in Fig. 9(b), it is still high enough to infer a strong positive association.

Figure 14(b) also shows a Perkes error grid for type 1 diabetes^40^ generated from the repeatability test results and the conversion coefficients obtained by the LLS fitting. Here, the color scale in the plot indicates the spatial density of nearby points. Although blood glucose values obtained by venous blood glucose testing or capillary blood glucose testing are generally used as the reference values for error grid analysis, CGM values were used as the reference values in this research. Because of this substitution, note that Fig. 14(b) has combined errors from the MI method and the CGM. Nevertheless, most of the data points in Fig. 14(b) are distributed between zones A and B. Specifically, ∼69% and 31% of the data are located in zones A and B, respectively, and there were no data points in other zones. This result compares favorably with some other previous non-invasive optical blood glucose monitoring research.^41^^,^^42^

### 3.3 Attempt to Improve the Accuracy of the Proposed MI Method

In this section, an attempt is made to improve the accuracy of the proposed MI method by combining the $L_{\mathrm{AC},\text{amplitude}}(t)$ correction.

In this study, the metabolic index MI shown in Eq. (32) has been used so far for a non-invasive BGL index. However, as shown in Eq. (31), the oscillation amplitude of deoxyhemoglobin $C_{\mathrm{Hb},\mathrm{AC}}(t)$ is also affected by $c_{0}$, L(t), and $L_{\mathrm{AC},\text{amplitude}}(t)$. Although the total hemoglobin concentration $c_{0}$ should be less susceptible to variation without drastic changes in body hydration level or acute anemia, the oscillation amplitude of the optical path length $L_{\mathrm{AC},\text{amplitude}}(t)$ and the optical path length L(t) may change depending on perfusion status, pressure, and other factors. In other words, BGL estimation based on a metabolic index MI may still be capable of better accuracy by taking L(t) and $L_{\mathrm{AC},\text{amplitude}}$ correction into account. However, in principle, it is impossible to measure the effective optical path length L(t) based on the MBLL. Therefore, the following assumptions and approximations are introduced.

Since it can be assumed that the AC amplitude of the optical path length $L_{\mathrm{AC},\text{amplitude}}(t)$ should increase as its counterpart $L_{\mathrm{LF}}(t)$ increases and that the divergence speed of $L_{\mathrm{AC},\text{amplitude}}(t)$ should not be greater than that of $L_{\mathrm{LF}}(t)$, the relationship between $L_{\mathrm{AC},\text{amplitude}}(t)$ and L(t) can be approximated as follows:

$$L_{\mathrm{AC},\text{amplitude}}(t)\propto {\left[L_{\mathrm{LF}}(t)\right]}^{n}\;(0<n\leq 1),$$ (33)

where n is an appropriate positive power exponent to associate $L_{\mathrm{AC},\text{amplitude}}(t)$ with $L_{\mathrm{LF}}(t)$ which is equal to or <1. By substituting Eq. (30) into Eq. (33), and by applying approximation of Eq. (14), the following relationship can be further derived.

$$\frac{L_{\mathrm{AC},\text{amplitude}}(t)}{L(t)}\approx \frac{L_{\mathrm{AC},\text{amplitude}}(t)}{L_{\mathrm{LF}}(t)}\propto \frac{L_{\mathrm{AC},\text{amplitude}}(t)}{{\left[L_{\mathrm{AC},\text{amplitude}}(t)\right]}^{\frac{1}{n}}},$$ (34)

$$={\left[L_{\mathrm{AC},\text{amplitude}}(t)\right]}^{1-\frac{1}{n}}\approx {\left[\frac{A_{{\mathrm{HbO}}_{2}}(t)+A_{\mathrm{Hb}}(t)}{c_{0}}\right]}^{1-\frac{1}{n}}.$$ (35)

Here, to make the rightmost term of Eq. (34) dimensionless, a dimensionless correction coefficient α is introduced as follows:

$$\alpha (t)={\left[\frac{A_{{\mathrm{HbO}}_{2}}(t)+A_{\mathrm{Hb}}(t)}{A_{{\mathrm{HbO}}_{2},0}+A_{\mathrm{Hb},0}}\right]}^{1-\frac{1}{n}}\;(0<n\leq 1),$$ (36)

where $A_{{\mathrm{HbO}}_{2},0}$ and $A_{\mathrm{Hb},0}$ are arbitrary-defined constant values that satisfy

$$A_{{\mathrm{HbO}}_{2},0}+A_{\mathrm{Hb},0}=c_{0}L_{\mathrm{AC},0},$$ (37)

where $L_{\mathrm{AC},0}$ is a constant standard optical length value for normalization. Assuming that $c_{0}$ can also be regarded as a constant value for each specific subject throughout a series of experiments, the correction coefficient α(t) should also be approximately proportional to $L_{\mathrm{AC},\text{amplitude}}(t)/L(t)$.

Finally, multiplying α(t) by MI(t) gives the following amplitude-corrected metabolic index ${\mathrm{MI}}^{\prime }(t)$

$${\mathrm{MI}}^{\prime }(t)=\alpha (t)\cdot \mathrm{MI}(t).$$ (38)

**Fig. 15:**
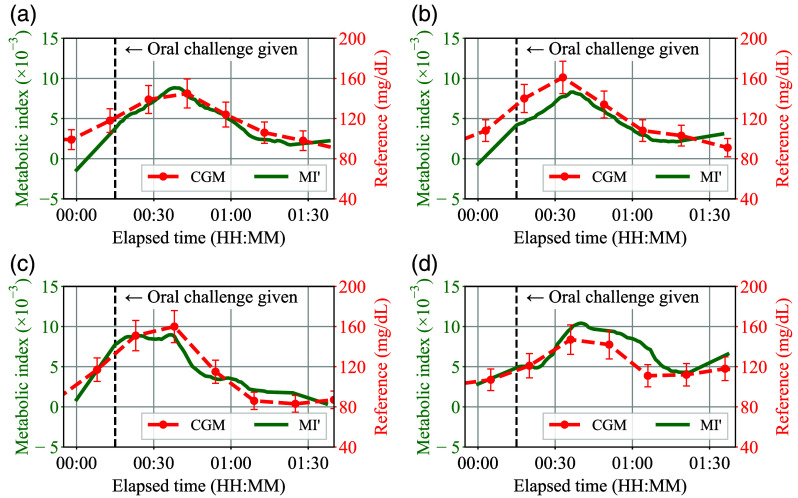
(a)–(d) Correction-applied evaluation results of the smartphone-based prototype corresponding to Figs. 13(a)–13(d), respectively.

Figures 15(a)–15(d) show the evaluation results of the α-corrected ${\mathrm{MI}}^{\prime }(t)$ obtained by the smartphone prototype, which correspond to and are plotted in the same manner as Figs. 13(a)–13(d), respectively. Note that n=0.5 of the power exponent was temporarily adapted in Eq. (36), which means that the AC amplitude of the optical path length is proportional to the square root of $L_{\mathrm{LF}}(t)$, and typical values of $A_{{\mathrm{HbO}}_{2}}(t)$ and $A_{\mathrm{Hb}}(t)$ in the resting state were chosen for $A_{{\mathrm{HbO}}_{2},0}$ and $A_{\mathrm{Hb},0}$. By comparing each plot in Fig. 15 with that of Fig. 13, it can be seen that the rise and fall in the metabolic index become clear with α correction. Among these, Figs. 13(c) and 13(d), which showed a moderate correlation with the CGM values, showed a better correlation in Figs. 15(c) and 15(d) by applying the correction.

**Fig. 16:**
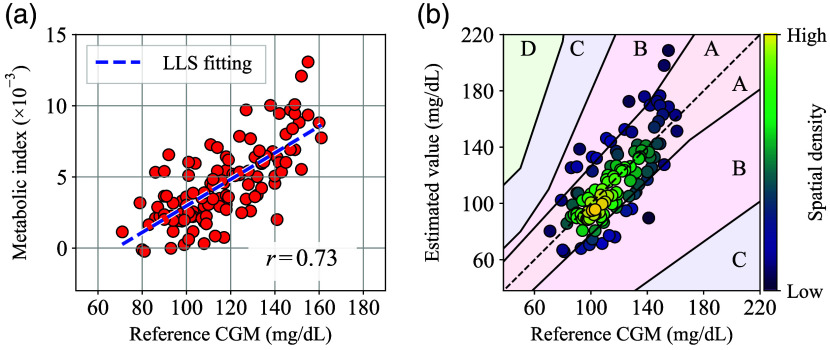
(a) Scatter plot of α-corrected metabolic index MI’ values versus reference CGM values generated from the repeatability test results. (b) Perkes error grid for type 1 diabetes generated from the α-corrected repeatability test results.

In addition, a scatter plot of the α-corrected metabolic index ${\mathrm{MI}}^{\prime }$ versus CGM values and a Perkes error grid for type 1 diabetes with α correction, generated based on the same experimental data set of Fig. 14, are shown in Figs. 16(a) and 16(b), respectively. Here, the same correction parameters were used as in Fig. 15. As can be seen in Fig. 16(a), the correlation coefficient r computed by the LLS fitting improved slightly to 0.73 with the α correction. In addition, in Fig. 16(b), ∼79% and 21% of the data points are distributed in zones A and B, respectively, and the zone A ratio has increased by 9% compared to Fig. 14(b).

According to these results of α correction, although n=0.5 of the power exponent is a tentative value and there is no biomedical basis to support this number yet, it can be seen that α correction can improve the MI-based non-invasive BGL estimation to some extent.

**Fig. 17:**
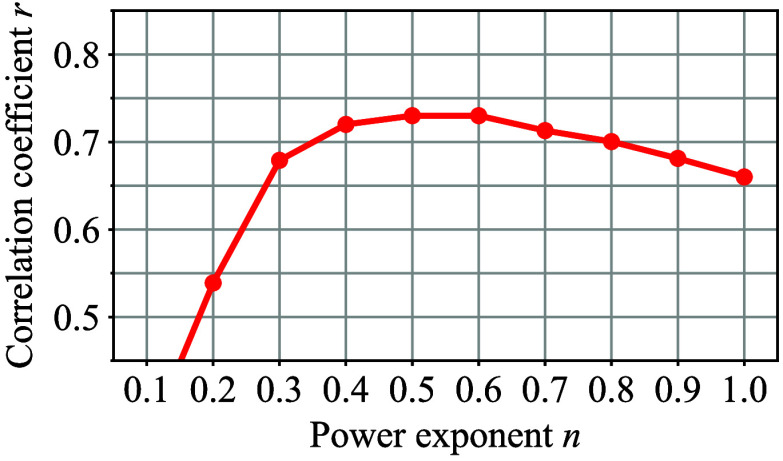
Transition of the correlation coefficient r computed by the LLS fitting according to different power exponents n.

As a reference, Fig. 17 shows a transition of the correlation coefficient r computed by LLS fitting after applying α correction for the same data set of the smartphone-based prototype according to different power exponents n. Here, the n=1 spot corresponds to the case without α correction. As can be seen from Fig. 17, r showed a gradual trend around n=0.4 to 0.7, which implies that the AC amplitude of the optical path length should be roughly proportional to $L_{\mathrm{LF}}(t)$ to the power of $\frac{1}{2}$ to $\frac{2}{3}$.

Finally, as a summary, Table 2 shows a comparison of the key performance indicators of BGL estimation with and without α correction. Here, the term MARD stands for mean absolute relative difference, which is the average of the error between the estimated value and the reference value divided by the reference value, and RMSE stands for root-mean-square error, which is the square root of the average of the squared errors between the estimated values and the reference values. MARD and RMSE are typical metrics used to evaluate the performance of glucose monitoring systems.^9^^,^^32^ Note that the absence of data other than in zones A and B is due more to an insufficient number of data points than to the good performance of the proposed MI method, and that MARD and RMSE here have combined errors from the MI method and the reference CGM.

**Table 2:** Comparison of key performance indicators of BGL estimation with and without α-correction.

	Without α-correction, data from Fig. 14(b)	With α-correction (n=0.5), data from Fig. 16(b)
Zone A percentage	69.2% (81/117 samples)	78.6% (92/117 samples)
Zone B percentage	30.8% (36/117 samples)	21.4% (25/117 samples)
MARD	17.5%	13.3%
RMSE	24.1 mg/dL	19.7 mg/dL

According to the manufacturer, all FreeStyle Libre^®^ CGM sensors in the United States have a MARD of <10%^34^ and a third-party evaluation has shown a roughly similar result.^43^ Although the proposed MI method, even with α correction, has an even larger MARD compared to the <10% MARD of the commercially available CGM, implementing hardware improvements and algorithm optimization may further reduce the MARD of the proposed method to below 10%.

For reference, Table S1 in Sec. S2 of the Supplementary Material shows the performance comparison between the proposed MI method and other non-invasive blood glucose monitoring methods.

## 4 Discussion

**Fig. 18:**
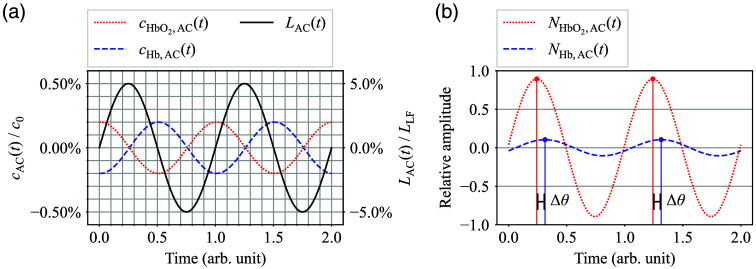
(a) Simplified plot diagram of the phase relationship between $c_{{\mathrm{HbO}}_{2},\mathrm{AC}}(t)$, $c_{\mathrm{Hb},\mathrm{AC}}(t)$, and $L_{\mathrm{AC}}(t)$. (b) Example of the AC component in NIRS signals calculated from the data in Fig. 18(a).

The proposed metabolic index MI was confirmed to show a good correlation with the reference CGM values, and an even better correlation by α correction. This result appears sufficient for supporting the assumption that there is a BGL-dependent small phase delay between oxy- and deoxyhemoglobin NIRS signals as a concept test. Looking at Eqs. (13) and (28) reveals that confirming the existence of the phase delay Δθ also means confirming that the relationship between optical path length pulsation $L_{\mathrm{AC}}(t)$ and oxyhemoglobin concentration pulsations $c_{{\mathrm{HbO}}_{2},\mathrm{AC}}(t)$ is approximately a sine and cosine relationship. For reference, Fig. 18(a) shows a simplified plot diagram of the phase relationship between $c_{{\mathrm{HbO}}_{2},\mathrm{AC}}(t)$, $c_{\mathrm{Hb},\mathrm{AC}}(t)$, and $L_{\mathrm{AC}}(t)$. Here, $c_{{\mathrm{HbO}}_{2},\mathrm{AC}}(t)$ and $c_{\mathrm{Hb},\mathrm{AC}}(t)$ are normalized to $c_{0}$, and $L_{\mathrm{AC}}(t)$ is normalized to $L_{\mathrm{LF}}$, and the relative amplitude of each plot was defined arbitrarily. Figure 18(b) shows an example of the AC component of NIRS signals calculated from the data in Fig. 18(a). Here, each curve is normalized to the total amplitude of both curves, and the markers in each curve indicate the local maxima, and the phase delay Δθ is shown in the plot. Figure 18(b) reveals that the phase delay Δθ can be generated numerically by combining $c_{{\mathrm{HbO}}_{2},\mathrm{AC}}(t)$, $c_{\mathrm{Hb},\mathrm{AC}}(t)$, and $L_{\mathrm{AC}}(t)$. Here, it should be noted that the magnitude of Δθ has been enhanced in Fig. 18(b) to enhance clarity. As shown in Fig. 9(c), an increase of 40  mg/dL in BGL results in a change of no >0.1 radians at Δθ magnitude, which is about 1.5% of a cardiac cycle. Here, the small degree of delay is assumed to have made it difficult to identify the presence in previous studies.

However, this study does not physiologically investigate why the amplitude of the deoxyhemoglobin pulsation increases with an increasing BGL which is the fundamental mechanism for estimating BGL by the MI method. This phenomenon of increasing deoxyhemoglobin pulsation may possibly stem from the glycolysis system, which links glucose to the metabolism. Since 2,3-bisphosphoglycerate (2,3-BPG) renders the effect of regulating the oxygen affinity of hemoglobin in the glycolysis system, 2,3-BPG may prove the key to revealing the mechanism behind the MI method. At the same time, oxidative phosphorylation could also potentially increase the deoxyhemoglobin amplitude by directly consuming oxygen from the blood. In future studies, direct observation of pure concentration pulsations in oxy- and deoxyhemoglobin, which eliminate the effect of optical path pulsation, and a comparison with BGLs and optical path pulsation should help to understand the basics of the MI method. Additionally, it is crucial to examine the influence of nutrients beyond carbohydrates on the MI. Table 1 indicates that the oral challenges utilized in this study lacked fat or protein. If oxidative phosphorylation is the driving force underlying MI, then amino acids and lipids could potentially exert an impact. Consequently, it is imperative to investigate protein and fat-rich oral challenge tests for a fundamental understanding of the MI method.

The MI method is also based on the assumption that the oscillation amplitude of the deoxyhemoglobin concentration $C_{\mathrm{Hb},\mathrm{AC}}(t)$ is proportional to the BGL. In other words, more oxygen is consumed with each cardiac beat as the blood glucose rises. In this sense, the MI method can be considered an optical version of the MHC method, using the correlation between the BGLs and the heat flux generated by metabolic oxidation. In addition, since the optical MI method should be less sensitive to environmental conditions and perspiration than the thermal-based MHC method, the MI method may probe more practical for wearable use.

The assumption behind the MI method may sound reasonable for healthy and non-diabetic individuals. However, for people with type 1 or type 2 diabetes, whose metabolic functions and glucose tolerance are abnormal, the relationship between the proposed MI and BGLs may change or even collapse in worst cases. On the other hand, given the fact that the evaluation result from the MHC method also shows a good correlation in high glucose concentrations of diabetic patients in the prior research,^15^ the MI method is still potentially applicable in high BGLs. Therefore, clinical testing in diabetic patients will serve as a key test of the proposed MI method. In addition, even among healthy people, there will be individual variations in MI-to-BGL calibration factors depending on gender, age, physical constitution, race, etc. In future research, the interdependence of these differentiators will need to be investigated.

In addition, even for a specific person, the daily variation of MI-to-BGL calibration factors must also be examined for the possibility of being affected by that person’s basal body temperature, ambient temperature, or other daily conditions. However, as a reference, in the case of the repeatability test mentioned in Sec. 3.2.1, although a series of tests extending over 1 month were performed, no significant deviation in the correlation weight between MI and BGL was confirmed at the beginning and end of the testing for a single test subject.

Next, looking at Figs. 9(b), 14(a), and 16(a), the lower BGL detection limit in the MI method is mainly considered as ∼70  mg/dL given that $C_{\mathrm{Hb},\text{amplitude}}(t)$ and MI(t) are always greater than or equal to 0. This estimated detection limit in the MI method is not a serious problem given the fact that a healthy human body tries to maintain BGLs above 70 mg/dL. However, for hypoglycemic patients, this limit can be fatal. Therefore, during actual operation of the MI-based glucose monitor in the future, rather than just displaying the estimated BGL, a low BGL alarm should be provided in case the reading falls below the threshold. The sensitivity or namely the minimum incremental detection limit should also be carefully investigated to ensure precise control of patient BGLs.

In addition, some kind of automatic data rejection for low-quality optical signals should be considered in future studies. In particular, from Eq. (36) it can be seen that α(t) and ${\mathrm{MI}}^{\prime }(t)$ increase as $A_{{\mathrm{HbO}}_{2}}(t)+A_{\mathrm{Hb}}(t)$ decreases, since $1-\frac{1}{n}$ is always less than or equal to zero while 0<n≤1. This implies the potential risk of detecting false hyperglycemia when the pulsation signal is poor. There is also a similar report that pulse oximetry may show inaccurate ${\mathrm{SpO}}_{2}$ values due to hypoperfusion.^44^ In fact, the authors found erroneous MI values at low room temperature or cold fingers during the preliminary experiment. The data rejection threshold for optical signals should therefore be properly set in order to avoid false estimations of BGL. Besides, as shown in Fig. 7, the raw MI data may contain noise as a result of signal distortion caused by body motion. To mitigate this issue, it is necessary to properly apply the body acceleration threshold, similar to the built-in heart rate and oxygen saturation monitoring features found on some smartwatches.

Furthermore, the inclusion of other physiological parameters in the MI method would be a viable option for robust BGL estimation in practical use. For example, in this study the total hemoglobin concentration $c_{0}$ was treated as a constant value. Obviously, $c_{0}$ strongly correlates with the hemoglobin content. Since non-invasive optical measurements of total hemoglobin content have been reported by other researchers,^45^ combining the estimated hemoglobin content with the proposed MI method may further improve estimation accuracy. The core body temperature and ambient temperature are also possible candidates for optional parameters. Conversely, combining the proposed metabolic index MI with previous research based on multivariate statistics or ML techniques may possibly improve their estimation accuracy.

Moreover, although all the oral challenge tests were performed in a sitting position in this paper, it is also necessary to investigate the potential impact of posture on the proposed MI method to ensure continuous daily use of this technique.

## 5 Conclusion

In this study, a method for non-invasive blood glucose estimation based on the phase delay between oxy- and deoxyhemoglobin was analytically derived. This phase delay-based metabolic index has not been reported by other researchers and is considered a scientifically important discovery. Then, the correlation between the proposed metabolic index MI and the BGLs measured by CGM has been confirmed by the smartwatch-based prototype and the repeatability for a single subject has been confirmed by the smartphone-based prototype, and about 69% of the data points are located in zone A according to the Perkes error grid analysis. Moreover, the possibility of improving the estimation accuracy by applying corrections based on the oscillation amplitude of the optical path length has been suggested.

In future studies, for the practical application of the proposed method, the personal and daily variation in the calibration factors of the proposed method, evaluation in unhealthy or diabetic subjects, and the correlation below 70 mg/dL of BGLs need to be investigated.

Since the proposed MI method can in principle be implemented in existing devices with a pulse oximetry function, and is inexpensive, battery-saving and simple compared with other non-invasive blood glucose monitoring methods using MIR spectroscopy or Raman spectroscopy, the MI method can be a powerful tool for portable BGL monitoring devices in the future. Moreover, the discovery that ordinary smartphones have the potential to serve as glucose meters could prove an enormous benefit to those individuals who are concerned about their glucose levels.

## Supplementary Material



## Data Availability

The data underlying the results presented in this paper are not publicly available at this time due to privacy and ethical concerns but may be obtained from the authors upon reasonable request.
