# Supplementary material for: Non-invasive blood glucose estimation method based on the phase delay between oxy- and deoxyhemoglobin using visible and near-infrared spectroscopy
Source: J Biomed Opt. 2024 Mar 5;29(3):037001. doi: 10.1117/1.JBO.29.3.037001 (PMC10913690; doi:10.1117/1.JBO.29.3.037001)
Supplement: Supplementary file 1 [file JBO_029_037001_SD001.pdf]

Supplementary Material

S1 Detailed process for formulating basic NIRS formulas

Figure 1 in the main article shows a schematic diagram of a NIRS measurement on a human body. Here, $I_{\text{in}}(\lambda, t)$ and $I_{\text{out}}(\lambda, t)$ are the incident and detected light intensities for the wavelength λ at time t, respectively. According to the modified Beer-Lambert law (MBLL), the attenuation of light intensity in a tissue can be expressed as follows:[21]

$$\text{OD}(\lambda, t) = \log_{10} I_{\text{in}}(\lambda, t)/I_{\text{out}}(\lambda, t) = A(\lambda, t) + S(\lambda, t), \tag{S1}$$

where $\text{OD}(\lambda, t)$ is the optical density for the wavelength λ at time t.
Eq. (S1) is the superposition of absorption $A(\lambda, t)$ and scattering $S(\lambda, t)$. Here, the molar concentrations of oxyhemoglobin and deoxyhemoglobin in the blood at time t, $c_{\text{HbO}_2}(t)$, $c_{\text{Hb}}(t)$ are the dominant light absorbers in the NIR region.[22] Therefore, the absorption of light in the NIR region can be defined as

$$A(\lambda, t) = \varepsilon_{\text{HbO}_2}(\lambda) \cdot c_{\text{HbO}_2}(t) \cdot L(\lambda, t) + \varepsilon_{\text{Hb}}(\lambda) \cdot c_{\text{Hb}}(t) \cdot L(\lambda, t), \tag{S2}$$

where $\varepsilon_{\text{HbO}_2}(\lambda)$ and $\varepsilon_{\text{Hb}}(\lambda)$ are the specific extinction coefficients of oxyhemoglobin and deoxyhemoglobin at the wavelength λ,[23] and $L(\lambda, t)$ is the optical path length with respect to the wavelength λ and the time t written as follows:

$$L(\lambda, t) = \text{DPF}(\lambda, t) \cdot d, \tag{S3}$$

where d is the distance between the light source and the photodetector and $\text{DPF}(\lambda, t)$ is the differential path length factor.[24, 25] Assuming that the scattering term $S(\lambda, t)$ is constant in Eq. (S1), the incremental OD value between the OD value at the time t and t_0 can be expressed as follows:

$$\Delta\text{OD}(\lambda, t) = \text{OD}(\lambda, t) - \text{OD}(\lambda_0, t_0) \tag{S4}$$

$$= \varepsilon_{\text{HbO}_2}(\lambda) \cdot \Delta(c_{\text{HbO}_2}(t) \cdot L(\lambda, t)) + \varepsilon_{\text{Hb}}(\lambda) \cdot \Delta(c_{\text{Hb}}(t) \cdot L(\lambda, t)). \tag{S5}$$

Assuming that the optical path length $L(\lambda, t)$ is nearly independent of wavelength in the limited spectral region, Eq. (S5) can be rewritten as

$$\Delta\text{OD}(\lambda, t) \approx \varepsilon_{\text{HbO}_2}(\lambda) \cdot \Delta(c_{\text{HbO}_2}(t) \cdot L(t)) + \varepsilon_{\text{Hb}}(\lambda) \cdot \Delta(c_{\text{Hb}}(t) \cdot L(t)). \tag{S6}$$

Here, for convenience, $\Delta(c_{\text{HbO}_2}(t) \cdot L(t))$ and $\Delta(c_{\text{Hb}}(t) \cdot L(t))$ are replaced by the oxy- and deoxyhemoglobin NIRS signals $N_{\text{HbO}_2}(t)$ and $N_{\text{Hb}}(t)$, respectively.

Additionally, from Eqs. (S1) and (S4), and assuming that $I_{in}(\lambda, t)$ is kept at the same value during the measurement, the incremental OD value ΔOD can be expressed as

$$\Delta\text{OD}(\lambda, t) = \log_{10} \frac{I_{\text{in}}(\lambda, t)}{I_{\text{out}}(\lambda, t)} - \log_{10} \frac{I_{\text{in}}(\lambda, t_0)}{I_{\text{out}}(\lambda, t_0)} = \log_{10} \frac{I_{\text{out}}(\lambda, t_0)}{I_{\text{out}}(\lambda, t)}. \tag{S7}$$

Finally, from Eqs. (S6) and (S7),

$$N_{\mathrm{HbO_2}}(t) = \Delta(c_{\mathrm{HbO_2}}(t) \cdot L(t)) = c_{\mathrm{HbO_2}}(t) \cdot L(t) - c_{\mathrm{HbO_2}}(t_0) \cdot L(t_0), \tag{1}$$

$$N_{\mathrm{Hb}}(t) = \Delta(c_{\mathrm{Hb}}(t) \cdot L(t)) = c_{\mathrm{Hb}}(t) \cdot L(t) - c_{\mathrm{Hb}}(t_0) \cdot L(t_0) \tag{2}$$

can be obtained by combining two different $\Delta\mathrm{OD}$ values obtained from two different wavelengths at the same time t, and by solving for

$$\begin{bmatrix} N_{\mathrm{HbO_2}}(t) \\ N_{\mathrm{Hb}}(t) \end{bmatrix} = \begin{bmatrix} \varepsilon_{\mathrm{HbO_2}}(\lambda_1) & \varepsilon_{\mathrm{Hb}}(\lambda_1) \\ \varepsilon_{\mathrm{HbO_2}}(\lambda_2) & \varepsilon_{\mathrm{Hb}}(\lambda_2) \end{bmatrix}^{-1} \begin{bmatrix} \log_{10}(I_{\mathrm{out}}(\lambda_1, t_0)/I_{\mathrm{out}}(\lambda_1, t)) \\ \log_{10}(I_{\mathrm{out}}(\lambda_2, t_0)/I_{\mathrm{out}}(\lambda_2, t)) \end{bmatrix}, \tag{S8}$$

where λ_1 and λ_2 are two suitable light source wavelengths that differ from each other.

S2 Performance comparison with other blood glucose estimation methods

Table S1 shows the comparison of performance metrics obtained in this study with those presented in previous research cited in this study.

Since performance metrics like RMSE and MARD are highly influenced by the glucose concentration range, and the total number of data points and test subjects differ in each research, it is not appropriate to compare these metrics directly across different studies. Furthermore, it is important to note that each value in the table represents only a typical value extracted from the abstract and conclusion of each cited article. For more detailed information, please refer to the references.

As mentioned in the Discussion section of the main article, oral challenge tests in diabetic patients will serve as a key test to examine whether the proposed MI method is comparable to preceding methods such as Raman spectroscopy, given the absence of data on higher BGL in this study.

Table S1 Comparison of performance metrics obtained in this study with those presented in previous research.

Technology	Ref.	Target Site	Type	Range	Accuracy
MIR laser	[7]	Palm	Non-invasive	80-160 mg/dL	Clarke Error Grid<br>Zone A: 84 %
Raman spectroscopy	[10]	Palm	Non-invasive	40-540 mg/dL	Parkes Error Grid<br>Zone A + B: 97.0 %,<br>MARD: 19.1 %,<br>RMSE: 34.2 mg/dL
PPG + ML	[19]	Finger	Non-invasive	50-400 mg/dL	Clarke Error Grid<br>Zone A: 87.7 %,<br>Zone B: 10.3 %
MHC	[17]	Finger	Non-invasive	50-540 mg/dL	Parkes Error Grid<br>Zone A + B: 93.9 %
CGM	[34]	Upper arm	Minimally invasive	40-500 mg/dL	Parkes Error Grid<br>Zone A + B: 99.9 %,<br>MARD: 9.2 %
This study (α-corrected)		Finger	Non-invasive	75-160 mg/dL	Parkes Error Grid<br>Zone A: 78.6 %,<br>Zone B: 21.4 %,<br>MARD: 13.3 %,<br>RMSE: 19.7 mg/dL

S3 Appendix

Table S2 shows a list of abbreviations used in the article.

Table S2 List of abbreviations

Abbreviation	Meaning
BGL	Blood Glucose Level
BPF	Band-Pass Filter
BPG	Bisphosphoglycerate
CGM	Continuous Glucose Monitoring
FFT	Fast Fourier Transform
ISF	Interstitial Fluid
LD	Laser Diode
LF	Low-Frequency
LLS	Linear Least Squares
MARD	Mean Absolute Relative Difference
MBLL	Modified Beer-Lambert Law
MHC	Metabolic Heat Conformation
MI	Metabolic Index
MIR	Mid-Infrared
ML	Machine Learning
NIR	Near Infrared
NIRS	Near Infrared Spectroscopy
PI	Perfusion Index
PPG	Photoplethysmography
RMSE	Root-Mean-Square Error
SMBG	Self-Monitoring Blood Glucose
